# Pathway landscapes and epigenetic regulation in breast cancer and melanoma cell lines

**DOI:** 10.1186/1742-4682-11-S1-S8

**Published:** 2014-05-07

**Authors:** Mariama El Baroudi, Dario La Sala, Caterina Cinti, Enrico Capobianco

**Affiliations:** 1 Laboratory of Integrative Systems Medicine (LISM), Institute of Clinical Physiology (IFC) & Institute of Informatics and Telematics (IIT), National Research Council (CNR), Pisa, 56124, Italy; 2 Department of Experimental Biomedicine and Clinical Neuroscience, Policlinico Universitario, Palermo, 90127, Italy; 3 Experimental Oncology Unit, Institute of Clinical Physiology (IFC), National Research Council (CNR), Siena, 53100, Italy; 4 Center for Computational Science, Miller School of Medicine, University of Miami, Miami, Florida 33136, USA

**Keywords:** Cancer, Demethylation, Pathway Landscapes, Regulatory Networks

## Abstract

**Background:**

Epigenetic variation is a main regulation mechanism of gene expression in various cancer histotypes, and due to its reversibility, the potential impact in therapy can be very relevant.

**Methods:**

Based on a selected pair, breast cancer (BC) and melanoma, we conducted inference analysis in parallel on a few cell lines (MCF-7 for BC and A375 for melanoma). Starting from differential expression after treatment with a demethylating agent, the 5-Aza-2'-deoxycytidine (DAC), we provided pathway enrichment analysis and gene regulatory maps with cross-linked microRNAs and transcription factors.

**Results:**

Several oncogenic signaling pathways altered upon DAC treatment were detected with significant enrichment. We represented the association between these cancers by depicting the landscape of common and specific variation affecting them.

## Introduction

Recent advances in the field of epigenetics have provided new insights on the global epigenetic modifications that promote cancer development and progression [1,2]. Notably, epigenetic therapy, which is a consequence of the reversible nature of the epigenetic changes that alter gene expression in many tumor histotypes [3,4], is the ultimate interest of many proposed research projects, including the present one. Among several drugs with anti-tumorigenic effect regulating the epigenetic status of cells, 5-Aza-2'-deoxycytidine (DAC, Dagogen) is a potent demethylating agent known for anti-leukemic effect in the mouse model [5,6]. DAC acts to correct epigenetic defect including reactivation of tumor suppressor genes (TSG) [7] silenced by epigenetic mechanisms in tumor tissues [2]. Combining DAC with a chemotherapeutic agent (Carboplatin) in patients with recurrent, platinum-resistant, Epithelial Ovarian Cancer (EOC), was shown to exert a potent demethylating effect justifying the need of further testing for clinical efficacy [8]. The DAC effect was evaluated in breast cancer (BC) cell lines by gene expression analysis: at the used concentrations, there is a role appearing for treatment in different cellular processes linked to *TNF-α*-dependent apoptosis [9]. Also, the safety of DAC combined with chemotherapy in metastatic melanoma was reported in [10].

Rationale for the association between BC and melanoma, is provided first of all by evidence: genetic relationships and common variant genes appeared in [11-13]; a high risk association of melanoma in BC patients was reported in [14]; this risk holds especially for patients not receiving anti-estrogen therapy [15]; several biomarkers have been proposed to identify cancer stem cells in these two tumors [16]. In general, risk factors such as family history play a role in both cancers. Moreover, carriers of mutations in BRCA2 - the BC predisposition gene - have an increased risk of melanoma, while carriers of mutations in the melanoma susceptibility gene - CDKN2A - exhibit a higher than expected risk of BC. We therefore hypothesized that pathways involved in the development of both cancers may to a certain extent overlap, and that survivors of one cancer may be prone to develop the other one. Specifically, epigenetic mechanisms which sum to mutations during cancer progression, can be explored through pathways that are in common between the two cancers and result up-regulated after DAC treatment.

Our goal is to investigate DAC treatment effects in MCF-7 (BC) and A375 (melanoma) cell lines. We elucidate pathway landscapes and regulatory networks following gene expression profiling and aimed to identify epigenetically modified genes. Once the functionally enriched pathways are identified, both transcriptional and post-transcriptional regulatory networks are derived with the ultimate goal of assigning to them a role of possible drivers of future developments in novel anticancer target therapies. The sections of the paper are organized into Methods, Results, and Discussion at the end.

## Methods

### Microarray analysis

The MCF-7 and A375 cell lines were cultured in DMEM medium supplemented with 10% fetal bovine serum, 2 mM L-glutamine, at split ratio of 1:4 twice a week. After 24 hours of spit the culture medium was changed with media containing 2,5 µM 5-Aza-2-dC (DAC). 5-aza-2'-deoxcytidine (Sigma) was prepared freshly prior of experiments as a 10 mM stock solution diluted in acetic acid: water (1:1). The treated cells were collected after 48 hours and the mRNA was extracted for pelleted cells. The steps for cDNA microarray analysis were mainly two: 1. Total RNA samples were isolated from treated/untreated cells using TRIZOL reagent (Invitrogen); 2. Concentration of purified RNA samples were determined by A260 measurement and the quality was checked by Lab-on-a-chip analysis (total RNA nano biosizing assay, Agilent) with the Agilent 2100 Bioanalyzer RNAs isolated from different tumor tissues, and transcribed in cDNAs, were used to carry out the analysis. The cDNAs from treated BC were labeled with cy5 red fluorescent dye and untreated BC with cy3 green fluorescent dye. Hybridization was done on a microarray chip called MWG Human Cancer Array containing 50-mer oligo probes for 1920 genes (1853 human genes associated with cancer, 27 control genes and 40 replicated genes). Spots of fluorescence intensity were read by dual laser scanner (BioDiscovery) and the values were processed with Mavi Pro-2.6.0. (MWG Biotech), by computing background subtraction, normalization to a number of housekeeping genes, and comparison with untreated cancers. In order to select deregulated genes, we considered the cy5/cy3 normalized ratio (NR), calculated for each gene and by taking the ratio of the intensity in cy5 (Ic5) and the normalized intensities in cy3 (nIc3). Then, to reduce variability, all ratio values were transformed in log base 2. For inclusion of highly deregulated genes, we considered as up-regulated or down-regulated genes those with *log2 (NR) > 2.0 *and *log2 (NR*) < -2.0, respectively.

### Functional enrichment and pathways analysis

The analysis of the list of the significantly deregulated genes is based on the F-Census database [17] (http://210.46.85.180:8080/fcensus/) in order to extract information from highly inconsistent cancer gene data sources including CGC (Cancer Gene Census), OMIM (Online Mendelian Inheritance in Man), AGCOH(Atlas of Genetics and Cytogenetics in Oncology and Haematology), CancerGenes, TSGDB (Tumor Suppressor Gene Database), TGDBs (Tumor Gene Family Databases), two lists (H-list and R-list) that report genes deregulated in cancer samples and identified by mutational screens of cancer genomes using high-throughput techniques, and post-transcriptional regulation predicted by some microRNA (miRNA) target prediction algorithms, including *TargetScan, PicTar, DIANA-microT *and *MirTarget2*. For bioinformatics and functional annotation, we used over-representation analysis (*ORA*), available in *ConsensusPathDB *[18] (http://cpdb.molgen.mpg.de/), and aimed at identifying the functional categories and biological pathways among the differentially expressed (DE) genes between treated and untreated cell lines in both cancers. ORA allows interactive querying to perform a functional enrichment (FE) from comparison of two lists of genes (deregulated genes versus the universe, i.e. the human genome). The output links the genes to the corresponding annotations, found in databases, i.e. KEGG, Biocarta, Reactome, wikipathways. Among these examples, the *ConsensusPathDB *latest version - release 26 - was used.

### Transcriptional and post-transcriptional regulatory network analysis

The detection of Transcription Factors (TFs) predicted to regulate the list of significantly deregulated genes upon DAC treatment in BC, was performed using *TFactS *[19] (http://www.tfacts.org/). *TFactS *DB contains genes responsive to TFs, according to experimental evidence reported in literature, and reports two datasets: (i) A sign-sensitive catalogue that indicates the type (up/down) of TF regulation exerted on its targets; (ii) A sign-less catalogue that includes all regulatory interactions contained in sign sensitive and further interactions without the specific type of regulation. *TFactS *takes as a query the two lists of up-/down-regulated genes and compares them with sign-sensitive catalog of manually curated annotated target genes, then returning the lists of activated and inhibited TFs whose annotated target genes show a significant overlap with the query genes.

We then merged the experimentally validated miRNA-target gene DBs: *miRTarBase V.3.5 *[20] (http://mirtarbase.mbc.nctu.edu.tw/), *miRecords V.3 *[21] (http://mirecords.biolead.org/), and *miR2Disease *[22] (http://www.mir2disease.org/), to build a non-redundant dataset of miRNA-target genes regulatory (human) interactions. We used this consensus dataset to predict the miRNA regulators among the list of significantly deregulated genes. With Cytoscape [23] (http://www.cytoscape.org/) we showed the transcriptional and post-transcriptional regulatory networks whose analysis required a tool called *"AdvancedNetworkMerged"*, merging networks by set operations (union, intersection and difference).

## Results

### Pathway signatures after DAC treatment

Overall, we have identified 335 DE genes in BC, and 195 DE genes in melanoma upon DAC treatment of MCF-7 and A375 cell lines. In particular, we found 221 up-regulated genes in BC and 111 up-regulated genes in melanoma based on log2 (NR) > 2, with 29 genes in common. Then, we found 114 and 84 down-regulated genes based on log2 (NR) < -2 in BC and melanoma, respectively, with 50 down-regulated genes in common, and 13 genes down-regulated in melanoma and up-regulated in BC. We integrated the epigenetically modified genes with the F-census database (see Additional File 1 (AF1) Table 1) and the information about their methylation state when available from previous work [24] centered on DNA methylation profiling in MCF-7 (Additional File 1 Table 2). The following examples are listed as top up-regulated genes in BC (Additional File 1 Table 1):

**CTAG1B **(cancer/testis antigen 1B) - is a member of cancer/testis (CT) antigens found expressed in normal testis and in many cancers [25,26]. We found CTAG2 up-regulated in BC, but down-regulated in melanoma. Thanks to the capacity of a subset of these antigens to activate a spontaneous cellular immune responses in cancer patients [27], they are ideal cancer antigen targets for tumor immunotherapy, especially for BC after DAC treatment as with adult T-cell leukemia/lymphoma (ATLL) [28];

**RAB30 - **is a member of Ras-associated binding proteins (Rabs), which are involved in regulating different steps during exocytosis [29]. In particular, Rab30 is Golgi-specific Rabs, required for the structural integrity of the Golgi apparatus [30], and it was found hypomethylated in MCF7, together with other up-regulated genes (Additional File 1 Table 2);

**MAGEA1 **and **MAGEB2 **- are members of CT antigens; in particular, MAGEA1 was found expressed in 10% of cancer cells in conjunctival melanomas [31], and could be another candidate working in combination with immunotherapy;

**FHL2- **is a member of the four-and-a-half-LIM-only protein family, found over-expressed in BC cell lines upon DAC [32]; it can inhibit the proliferation and invasive growth of human breast cancer cells by repressing the functional activity of an inhibitor of DNA binding 3 (ID3) [33].

The following examples are listed as top up-regulated genes in melanoma (Additional file 1 Table 1):

**S100A2 **- is an EF hand calcium binding protein A2, given that the S100 gene family includes at least 13 members that are involved in the regulation of a number of cellular processes such as cell cycle progression and differentiation. It is a potential biomarker in several cancers including melanoma and BC, and may have a tumor suppressor function [34]. Its expression is stimulated by Jun transcription factor which was found up-regulated upon DAC treatment in both cancers;

**IGF2 **(insulin-like growth factor 2) - is up-regulated in BC. It was recently shown that the epigenetic alterations of IGF-2 are associated with development and progression of hepatocellular carcinoma (HCC) [35];

**FANCA **(Fanconi anemia, complementation group A) - mutations in this gene represent the most common cause of Fanconi anemia. There are 15 known Fanconi anemia genes involved in different pathways that coordinate multiple DNA repair [36].

The following genes commonly down-regulated in both cancer cell lines after DAC were found:

**CDKN2B - **is a cyclin-dependent kinase inhibitor 2B (known also as p15, and it inhibits CDK4) and a key regulator of biological processes repressing cell cycle progression by inhibition of cdk4 and cdk6 [37]. This gene is known as a melanoma susceptibility gene and mutations carrier in CDKN2A, correlated with a higher risk of BC. Since down-regulation upon DAC treatment results in both cancer cell lines, it may represent a side effect of treatment. The balance between DNA methylation and demethylation is a critical regulator of the methylation status of cyclin-dependent kinase inhibitor [38]. In fact, it is known that the demethylating agents function as DNA methyltransferase inhibitors, can be incorporated into the genome during DNA replication, and bind DNA methyltransferases that have the catalytic domain. This may lead to global hypomethylation and re-expression of both tumor suppressor genes and proto-oncogenes misregulation [39];

**CDH6 **(cadherin 6) - is a membrane glycoprotein and a member of the cadherin superfamily type II involved in cell-cell adhesion, differentiation and morphogenesis. The aberrant expression of cadherin-6 correlates with a poor prognosis in patients with E-cadherin-absent Renal cell carcinomas (RCC) and could be a useful tool to estimate the malignancy potential [40];

**CDH7 **(cadherin 7) - is another member of the cadherin superfamily type II. It was shown that CDH7 plays a role in tumor development of malignant melanoma cells by interacting with melanoma inhibitory activity protein (MIA) and migration melanoma cell [41].

The top down-regulated genes in the A375 cell line are listed below:

**BAI1 **(brain-specific angiogenesis inhibitor 1) - is a tumor suppressor gene and transmembrane protein with anti-angiogenic and antiproliferative activity [42];

**FKSG2 **- is a tumor protein, translationally-controlled 1 pseudogene, which we found down- regulated in BC too.

We found discrepancy in the regulation of some genes upon DAC treatment, with evidence of genes down-regulated in melanoma and up-regulated in BC. Examples worth of explanation are the following:

1. **CFLAR **- is a CASP8 and FADD-like apoptosis regulator, and structurally similar to caspase-8. It is found expressed in pulmonary metastases in osteosarcoma patients and human xenografts [43]. Its down-regulation contributes to apoptosis in human lung cancer cells, suggesting that its targeting may represent a promising therapeutic strategy for the cancer patients with over-expression of this gene [44];

2. **MDM2 **(Mouse double minute 2) - is an oncogene E3 ubiquitin protein ligase, regulated by p53 in case of DNA damage and part of an autoregulatory negative feedback loop [45];

3. **PRKCH **(protein kinase C, eta) - is a calcium-independent and phospholipids-dependent protein kinase. It is mostly expressed in epithelial tissues and has been shown to activate the protein kinase cascade that targets CCAAT/enhancer-binding protein alpha (CEBPA), following the same expression as PRKCH in both cancer cell lines [46].

**Figure 1:**
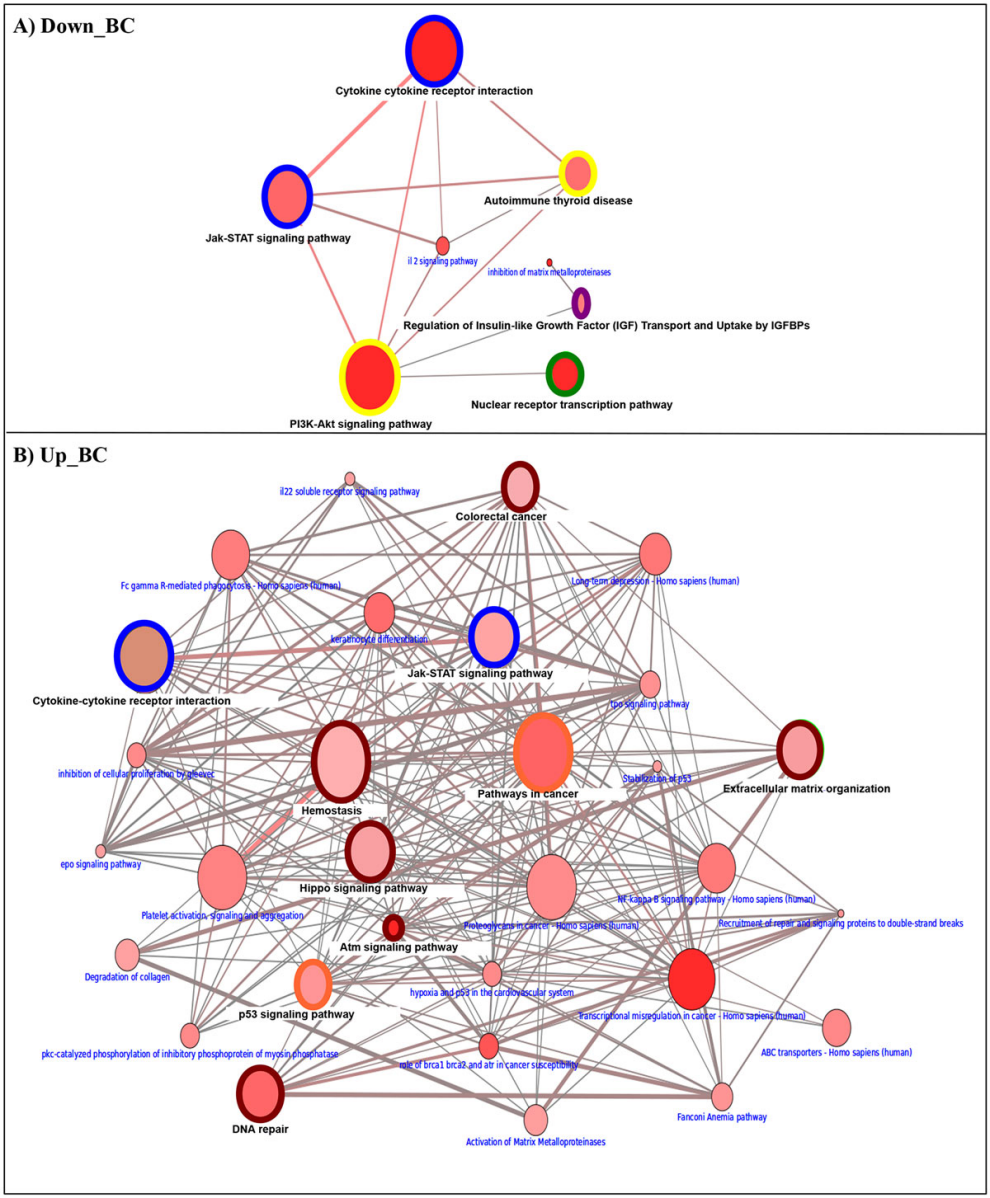
**Breast cancer and integrated Melanoma pathway landscape**. The *color node border *indicates the common pathways among differentially expressed genes in two cancer cell lines: Green for down-regulated in both tumors; Dark red for up-regulated genes in both tumors; Blue for up/down-regulated in BC and down in melanoma; Yellow for down-regulated in BC and up/down-regulated genes in melanoma; Orange for up-regulated and down-regulated genes in BC and melanoma, respectively and pink color for down-regulated and up-regulated genes in melanoma and BC, respectively. **A) **Enriched pathways among down- regulated genes in BC; B) Enriched pathways among up-regulated genes in BC.

**Figure 2:**
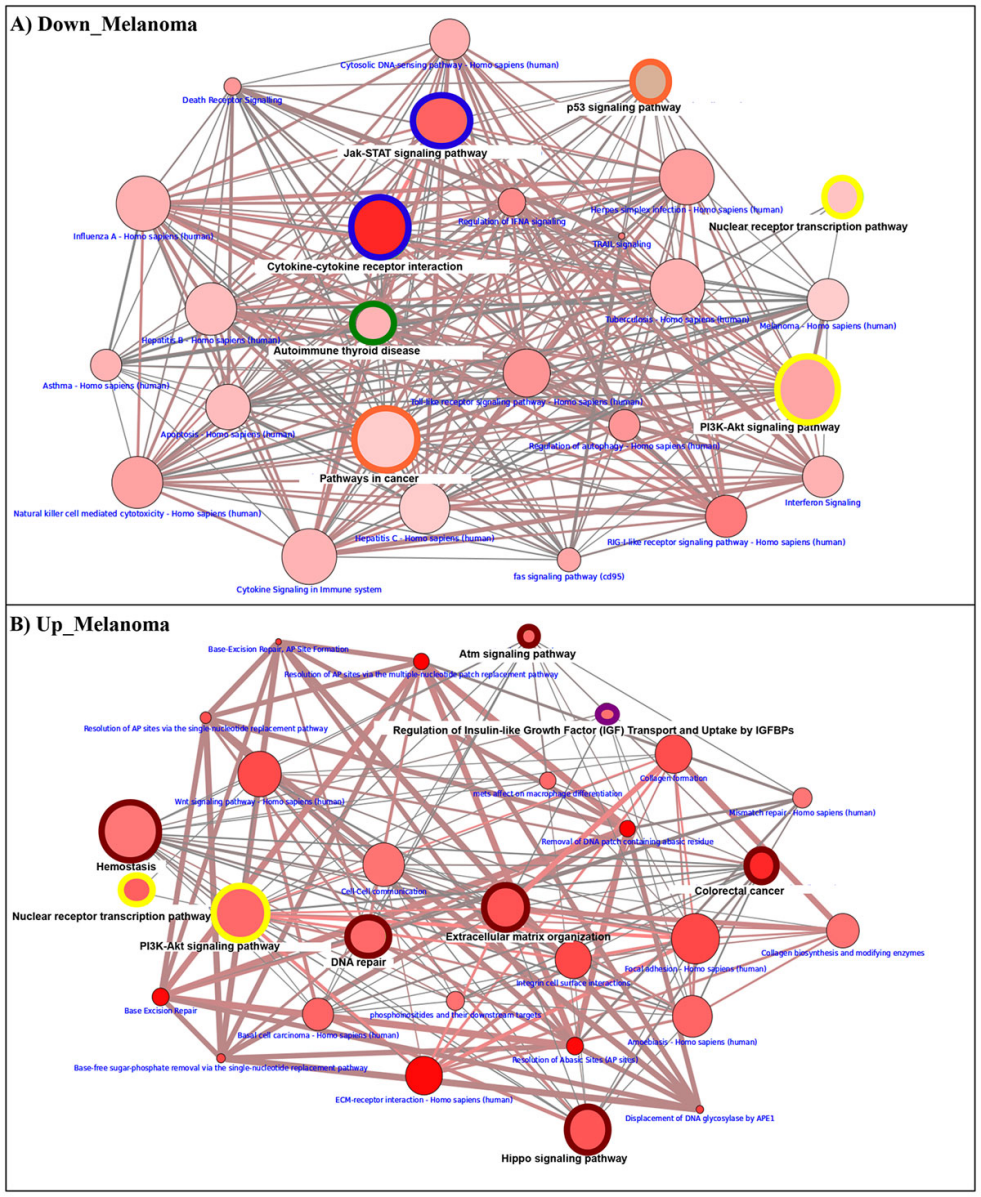
**Melanoma and integrated Breast cancer pathway landscape**. The *color node border *indicates the common pathways among differentially expressed genes in two cancer cell lines: Green for down-regulated in both tumors; Dark red for up-regulated genes in both tumors; Blue for up/down-regulated in BC and down in melanoma; Yellow for down-regulated in BC and up/down-regulated genes in melanoma; Orange for up-regulated and down-regulated genes in BC and melanoma, respectively and pink color for down-regulated and up-regulated genes in melanoma and BC, respectively. A) Enriched pathways among down- regulated genes in melanoma. B) Enriched pathways among up-regulated genes in melanoma.

Using ORA from the consensusPathDB, we found a list of over-represented functional categories and pathways referring to up- and down-regulated genes in the cancer cell lines. The enriched pathways are reported in Additional File 1 Table 3. The graphical tool in consensusPathDB was used to select the most interesting pathways involved in up- and down-regulated genes (Figure 1and Figure 2). The node size indicates the size of the gene set; the node color corresponds to the p-value (deeper red means smaller p-value); the edge color represents the number of shared genes in the predefined dataset; the color node border indicates the common pathways among genetically regulated genes. In Figure 1 and Figure 2 we show the common pathways regulated upon DAC treatment; in Table 1 we report the common enriched pathways among up-regulated genes.

**Table 1:** The common pathways associated to up-regulated genes.

Type CCL	Pathway name	Genes	p-value	q-value
BC	Atm signaling	ATM; MDM2; RBBP8; JUN; TP73	2.81E-06	7.96E-04
Melanoma		JUN; TP73	5.73E-03	3.42E-02
BC	Colorectal cancer	JUN; TGFB3; RAF1; DCC	9.04E-03	6.89E-02
Melanoma		MSH2; JUN; DCC; TCF7L2	6.36E-04	1.45E-02
BC	DNA repair	XPC; LIG3; ATM; TDG; ERCC6; FANCE; FANCC; RAD50	1.30E-04	9.17E-03
Melanoma		MUTYH; POLD1; TDG; FANCA	5.94E-03	3.42E-02
BC	Extracellular matrix organization	MMP14; TGFB3; ADAM17; CTSG; COL1A2; MMP8; CTSL2	4.01E-03	5.02E-02
Melanoma		COL3A1; COL1A2; ITGB4; TIMP1; COL1A1	2.78E-03	2.38E-02
BC	Hemostasis	IGF2; PRKCH; PPP2R1B; TGFB3; AKAP1; SERPINC1; VAV2; PRKCE; ANGPT2; GNA12; GNA13; ABCC4; RAF1	9.01E-03	6.89E-02
Melanoma		IGF2; TIMP1; SERPINC1; CD47; PRKCE; GRB7; PDPK1; ITPR3	9.42E-03	4.30E-02
BC	Hippo signaling	WNT2B; PPP2R1B; TGFB3; WNT1; ID2; TP73; TEAD4	4.62E-03	5.02E-02
Melanoma		DVL3; LATS1; FZD4; TP73; TCF7L2	3.11E-03	2.50E-02

The commonly enriched pathways associated to up-regulated genes is **ATM signaling **(Ataxia telangiectasia mutated) which is associated with the activation of p73, a member of the p53 tumor suppressor family regulating cell cycle and apoptosis after DNA damage, and found inactivated in many tumor histotypes [47,48]. The methylation status of p73 was shown to be common in patients with MDS and associated with poor prognosis [49]. p73 plays also a key role in p53 signaling pathway exerting an anticarcinogenic effect [50]; the activation of this signaling pathway induced by a natural diterpenoid compound was associated to G(2)/M cell cycle arrest in human melanoma cells to repair DNA damage. Similarly, such mechanism could be activated here, due to the up-regulation of genes involved in DNA repair (e.g. ATM is an important cell cycle checkpoint kinase). Then, evidence includes XPC that plays a central role in the recognition of DNA damage; LIG3 which is involved in excision repair; TDG which is important in DNA demethylation via the base excision repair pathway [51], in cellular defense against genetic mutation, in regulation of the epigenome and gene expression in non-melanoma skin cancer [52]; MUTYH that is involved in oxidative DNA damage repair in mammalian cells [53]. Another interesting pathways activated in both cancer cell lines is the extracellular matrix organization which involves up-regulation of different collagen genes like (COL1A1, COL1A2, COL3A1), and matrix metallopeptidase like (MMP8, MMP14).

Table 2 lists the common pathways among down-regulated genes, and also the genes involved in each specific pathway. We found also Nuclear receptor transcription pathway enriched among down-regulated genes like NR1D1, which is a member of the nuclear receptor subfamily 1, also found expressed in human endometrial stromal and epithelial cells [54].

**Table 2:** The common pathways associated to down-regulated genes.

Type CCL	Pathway name	Genes	p-value	q-value
Melanoma	Cytokine-cytokine receptor interaction*	CSF2RB; IL2RA; TNFSF18; TNFSF15; IFNA1; IL13; IL4; IL12A; IFNB1; TNFRSF10B; BMPR1B; IFNA8; IFNA5; TNFRSF18; LIFR	3.63E-11	4.24E-09
BC		IL2RA; IFNA8; IFNA1; BMPR1B; TNFSF8; IL2; IL26; TNFRSF18	2.92E-04	1.61E-02
Melanoma	Jak-stat signaling pathway*	CSF2RB; IL2RA; IFNA8; IFNA1; IL13; IFNA5; IFNB1; IL4; IL12A; LIFR	3.09E-08	1.81E-06
BC		IL2RA; IFNA1; IFNA8; IL2; IL26	3.41E-03	6.86E-02
Melanoma	PI3K-AKT signaling pathway#	IL2RA; IFNA8; IFNA1; IFNA5; IFNB1; IL4; FGF12; MDM2; FGF20	2.11E-04	1.76E-03
BC		IL2RA; IFNA8; IFNA1; THBS2; RXRA; IL2; FGF12; TSC2; FGF20	3.39E-04	1.61E-02
Melanoma	Nuclear receptor transcription pathway#	NR1D1; AR; NR2E1	3.84E-03	1.36E-02
BC		NR1D1; NR2E1; RXRA; RORB	3.43E-04	1.61E-02
Melanoma	Autoimmune thyroid disease	IL4; IFNA8; IFNA1; IFNA5	2.71E-04	1.98E-03
BC		IFNA1; IFNA8; IL2	4.56E-03	7.15E-02

Some of the listed pathways are associated to up-regulated genes in BC (*) and in melanoma (#).

In Table 3, we reported pathways that we found commonly enriched among up-regulated genes in one cancer cell line and down-regulated genes in the other cell line. The first example is the p53 signaling pathway, associated to up-regulated genes in MCF7. This is the case of PPM1D, a protein phosphatase, Mg2+/Mn2+ dependent, 1D, found over-expressed in different types of human tumors with poor prognosis, and negatively regulating p38 MAPK activity to reduce the phosphorylation of p53 [55,56]. PPM1D could dephosphorylate ATM and MDM2 [57] involved in this pathway. Another key regulator in this pathway is TNFRSF10B (tumor necrosis factor receptor superfamily, member 10b), involved in apoptosis transduction [58]. Then, regulation of insulin-like growth factor (IGF) transport and uptake by IGFBPs that involves up-regulated genes in A375, e.g. IGF2 (insulin-like growth factor 2, also called somatomedin A), implicated in growth and development. It is known that epigenetic alterations of IGF2 is correlated with severity of hepatocellular carcinoma development and progression. Additionally, down-regulated genes are involved, e.g. MMP2, which is a metalloproteinase implicated in cancer progression and metastasis whose increase in expression is associated with a poor prognosis [59,60] .

**Table 3:** The rest of common pathways associated to up or down-regulated genes in both cancer cell lines.

Gene expression	Pathway name	Genes	p-value	q-value
Up-BC	P53 signaling pathway	ATM; MDM2; PPM1D; APAF1; TP73	2.01E-03	4.56E-02
Down-melanoma		TNFRSF10B; MDM2; CASP8; BAI1	7.57E-04	4.12E-02
	Pathways in cancer	CDKN2B; CEBPA; CASP8; AR; FGF12; MDM2; FGF20	3.65E-03	1.33E-02
Up-BC		PGF; PTGS2; CEBPA; TGFB3; WNT1; JUN; DCC; STAT5B; CSF2RA; WNT2B; MDM2; RAF1; PAX8; IL8	1.05E-04	9.17E-03
Up-melanoma	Regulation of insulin-like growth factor (IGF) transport and uptake by IGFBPs	IGF2; IGFBP6	7.77E-03	4.09E-02
Down-BC		IGFBP1; MMP2	8.00E-03	9.59E-02

### Transcriptional and post-transcriptional regulatory networks

**Figure 3:**
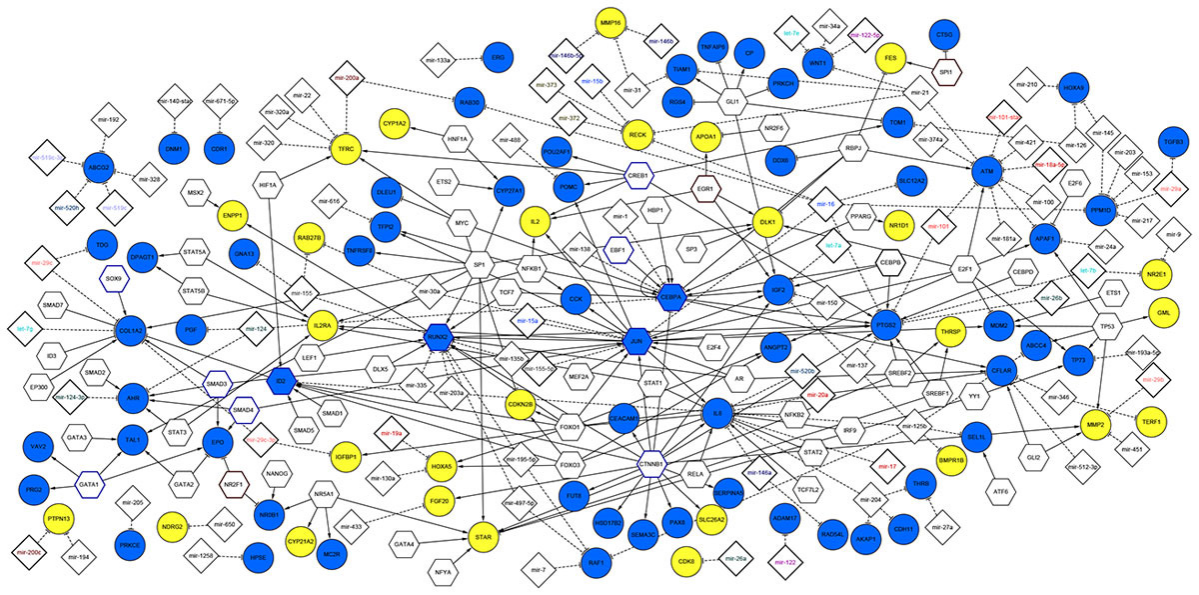
**The global network resulted from the union of transcriptional and post-transcriptional networks in MCF7**. *Node color*: blue for up-regulated genes while yellow for down-regulated genes; *Node border color*: dark brown indicates the significant TFs inhibited and blue for TF activated among differentially expressed genes (with p-value<0.05) and the non-colored nodes correspond to TFs that regulate at least one target of the epigenetically modified genes from TFactS analysis.

**Figure 4:**
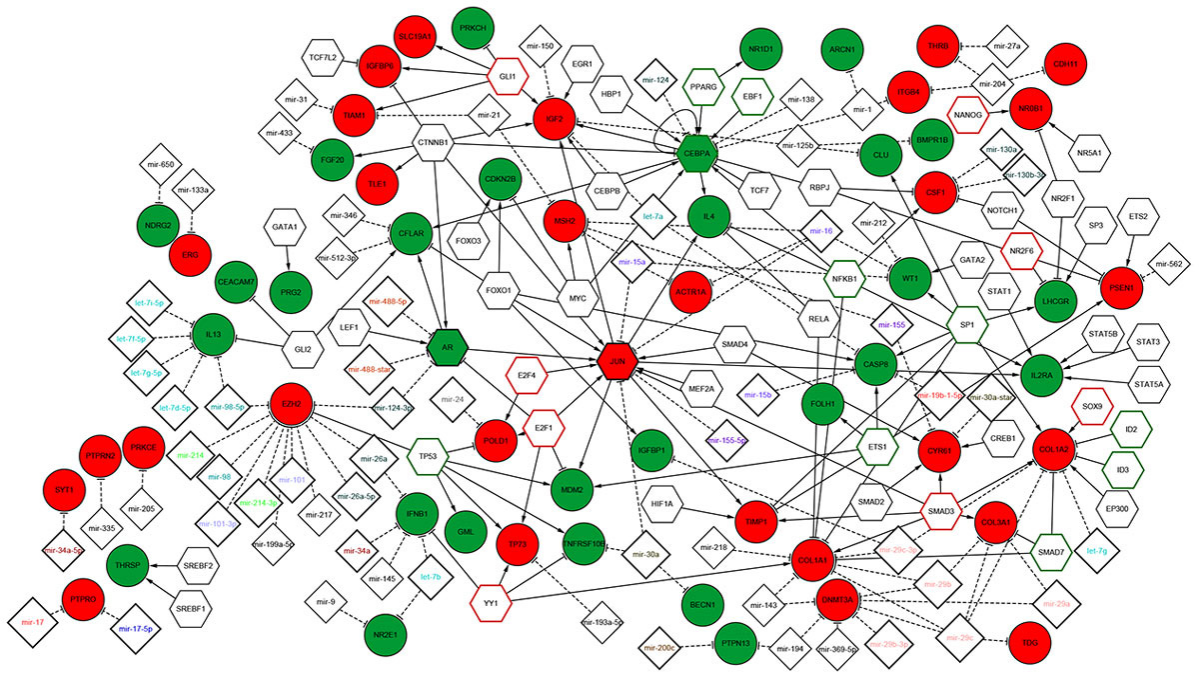
**The global network resulted from the union of transcriptional and post-transcriptional networks in A375**. *Node color*: red for up-regulated genes while green for down-regulated genes; *Node border color*: green indicates the significant TFs inhibited and red for TF activated among differentially expressed genes (with p-value<0.05) and the non-colored nodes correspond to TFs that regulate at least one target of the epigenetically modified genes from TFactS analysis.

**Figure 5:**
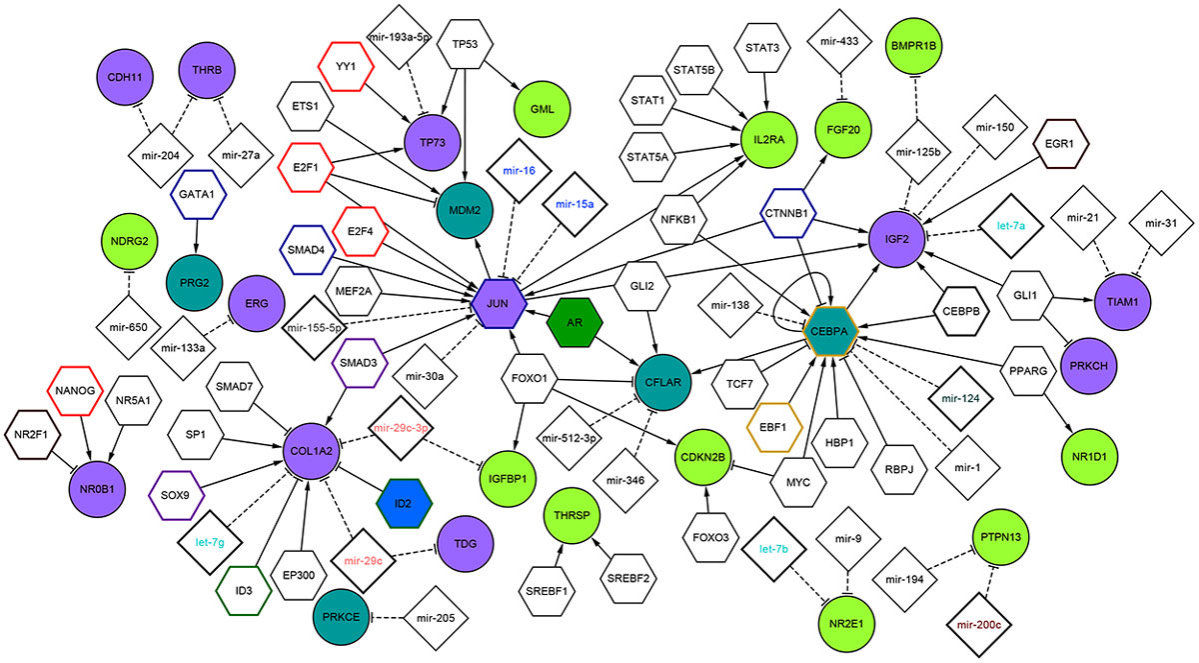
**The sub-network resulted from the intersection of regulatory map in MCF7 and A375: *Node color*: pink for up-regulated genes and light green for down-regulated genes common in two cancer cells, turquoise color to indicate genes up-regulated in BC and down-regulated in melanoma**. *Node border color*: we used pink to show the common activated gene and brown if the TF is inhibited in one cell and activated in the other cell. We keep the same border color from figure 3 and 4 if TFs are common but are signifivantly enriched only in a specific cell lines.

We reported in Additional File 1 Table 4 the TFs that were found significantly enriched among the deregulated genes by using the *TFactS *analysis tool. We built with *"AdvancedNetworkMerged" *in Cytoscape the union of transcriptional network and the miRNA regulatory network to show TFs and miRNAs experimentally validated as candidate regulators of the epigenetically modified genes in BC (Figure 3) and melanoma (Figure 4). Then, we extracted the sub-networks resulting from the intersection of the two regulatory networks (Figure 5). As a result, the epigenetic profile is described in an integrative network context.

In Figure 3, we reported the regulatory map of DE genes in MCF7. This network is composed by 300 edges, 242 nodes that correspond to 66 up-regulated and 28 down-regulated genes, 67 predicted TFs and 85 miRNAs. Among the predicted TFs, we found four - **CEPBA, JUN, RUNX2 **and **ID2**- that are up-regulated upon DAC treatment. In Figure 4, we reported the regulatory map of the epigenetically modified genes in A375 which is composed by 218 edges, 173 nodes matching 28 up-regulated and 29 down-regulated genes, 53 predicted TFs and 66 miRNAs. Among the predicted TFs we found three - **CEPBA, JUN **and **AR**- that are deregulated upon DAC treatment. In Figure 5, we reported the sub-network resulted from the intersection of the two regulatory networks in MCF7 and A375. This network is composed by 117 edges; 94 nodes that correspond to 12 up-regulated, and 11 to down-regulated genes in both cells, while 4 are up-regulated in BC and down-regulated in melanoma. Then, there are 41 predicted TFs and 28 miRNAs.

We found that the common up-regulated TF in both cell lines is **JUN**, a protoncogene that plays a dual role in regulation of tumor cell proliferation [61], and recently reported as candidate biomarker for personalized therapy in thymic epithelial tumors [62]. Its up-regulation is associated to the activation of IGF2 in both cell lines, and to an increased expression of MDM2 in MCF7. JUN represents a target of different miRNAs. For instance, **miRNAs-15a/16 **cluster, considered a tumor suppressor found deleted in different malignancies [63,64], or **miR-155 **reported as repressor of JUN in human dermal fibroblasts in vitro [65]. Interestingly, the up-regulation of Jun TF in MCF7 is connected to the activation of TIMP1, a mettallopeptidase inhibitor whose overexpression contributes to antimetastatic effect in BC [66], and to the activation of NQO1 (NADPH quinone oxidoreductase 1), playing a cytoprotective role and found associated to increase cell-sensitivity to BC anticancer treatment [67].

An important JUN target is the **SERPINB5 **(serpin peptidase inhibitor clade B, member 5), found also up-regulated upon DAC treatment in MCF7, and known as an important suppressor of the invasion and migration of cancer cells [68]. Then, another target is **STAT5B **(signal transducer and activator of transcription 5B), a member of the STAT family of TFs, normally activated in response to cytokines and growth factors signals. It has been shown that the regulation of STAT1/STAT5 signalling pathway mediated by STAT5B is important in the regulation of essential functions in the mammary gland [69]. The TFact tool reported among the up-regulated target of STAT5B the DPAGT1 target, which is an enzyme that catalyzes glycoprotein biosynthesis and was found to be targeted also by wnt/β-Catenin signalling pathways, mediating a variety of critical developmental processes [70].

Another important key regulator is **CEBPA**, which is down-regulated in melanoma and up-regulated in BC, a key TF involved in the regulation of cellular processes, especially in Hematopoietic system [71]. It was found epigenetically modified and post-transcriptionally regulated by miR-124 after DAC and trichostatin treatment in AML [72]. The repression of CEBPA by miR-138 was found to inhibit adipogenic differentiation of human adipose tissue-derived mesenchymal stem cells [73]. The post-transcriptional mechanism of regulation may be the same also in both cancer cell lines (Figure 4). CEPBA, being up-regulated in MCF7, cooperates with RUNX2 (runt-related TF 2) to activate **PTGS2 **(prostaglandin-endoperoxide synthase 2). The latter is known as cyclooxygenase-2 (COX-2), an inducible enzyme responsible in the prostanoid biosynthesis and involved in inflammation and mitogenesis [74]. PTGS2 is a target for development of anticancer therapy, and the use of anti-inflammatory PTGS2 inhibitors represents a promising strategy in the treatment of solid tumors [75]. In the A375 cell line, the down-regulation of CEPBA could be responsible of the up-regulation of PSEN1 (presenilin 1), mutated in patient with sporadic early-onset Alzheimer's disease [76].

An important up-regulated gene in the A375 cell line is **EZH2 **(Enhancer of zeste homolog 2), a member of polycomb protein and a part of polycomb repressive complex, implicated in cancer stem cell maintenance and metastasis in breast and pancreatic cancer [77,78], and it could be considered as a prognostic marker in renal cell carcinoma [79]. EZH2 is repressed by several miRNAs. It was reported in [80] that the loss of miR-101 expression correlates with the increase in EZH2 expression in invasive squamous cell carcinoma.

## Discussion

An accurate curation of pathway database with and regulatory relations was our preliminary step to draw a representative map of the major gene regulators and pathway landscapes involved in the DAC treatment of the MCF7 breast and the A375 melanoma cell lines. Then, a dissection of the key pathways and the knowledge of the interconnectivity between its components have been computed because conceived as valuable knowledge to gain towards new therapeutic approaches targeting the common genes involved in oncogenic pathways activated upon DAC treatment.

Our results are in agreement with previous studies showing the mechanism underlying the anticancer effect of DAC treatment, and its involvement in activation of the ATM signaling pathways in cancer cell lines [81]. The identification of a potential TF-miRNA regulatory network contributes to shed light on the potential molecular mechanism played by DAC treatment in both cancer cells. It is important to notice that DAC could have a different effect in the regulation of key regulators depending on the cancer cell line. One of the controversial molecular mechanisms is the increase of CEBPA expression in MCF7 and its down-regulation in A375. However, its post-transcriptional regulation by miR-124 results affected by demethylating agent, suggesting that the effectiveness of epigenetic therapy already shown in AML could be further investigated in both the cancer cell lines examines here.

Notably, some other interesting aspects are worth deeper discussion. The DAC treatment in MCF-7 and A375 cells induces down-regulation of different cytokines and interferons genes that represent key regulators of the cytokine-cytokine receptor interaction, Jak-STAT and PI3K-AKT signaling pathway, and they are associated to autoimmune thyroid disease. IL2RA and IL-2 are the common genes among all these pathways. The up-regulation of the interleukin-2 (IL-2) and its receptor alpha (IL2RA) are associated with the malignancy of the infiltrating human breast cancer. It may be the case that their down-regulation could produce an anticancer effect. The deregulation of these cytokines and interferons genes may suggest a potential role as breast cancer biomarker, or their possible use for novel DAC-combined treatments.

All these evidences might help the design of new therapeutic formulations, and the identification of the best therapeutic combinations between the use of demethylating agent as DAC and immonotherapy, e.g directly targeting the CT antigens, the cancer antigens, or other therapeutic tools that could improve the anticancer effect of the demethylating agent, obtaining therefore more efficient BC therapy. Moreover, we are confident that gene expression and pathway signatures could give aid to the discovery of relevant connections among molecular mechanisms and drugs so as to significantly contribute to the improvement of drug for cancer therapy. As a follow up agenda for future research, we are planning to extend our analysis to other tumor histotypes, and also to use novel gene expression profiling (exploiting transcriptome landscapes from RNA-Seq) for refinement of pathway signatures.

## Competing interests

The authors declare that they have no competing interests.

## Authors' contributions

ME participated in design of the study, performed the bioinformatic analysis and wrote the paper. DL carried out the microarray experiments. CC design and coordination of the study. EC design and coordination of the study and wrote the paper. All authors read and approved the final manuscript.

## Supplementary Material

Additional File 1 **AF1 Table 1: **The list with the expression values of deregulated genes in both cancer cell lines and the corresponding information extracted from F-census about Gene type: oncogene or TSG (tumor suppressor gene) extracted from both CGC and TSGDB; cancer type according to the classes provided in COSMIC; mutation frequency calculated by six high-throughput mutational screen data of cancer genomes; the number of miRNA predicted to regulate the target cancer genes, extracted from prediction tools including TargetScans, PicTar, DIANA-microT and MirTarget2. **AF1 Table 2: **Additional information for deregulated genes in MCF7 provided from detection of DNA methylation state in BC. **AF1 Table 3: **Enriched Gene Ontology Biological processes (GO BP) associated to discordant DE genes. **AF1 Table 4: **The list of the most enriched functional categories and pathways among the epigenetically modified genes in both cell lines extracted from ConsensusPathDB using ORA tool. **AF1 Table 5: **The list of TFs found significantly enriched among the epigenetically modified genes in BC and Melanoma. **AF1 Figure 1: **Graphical representation of the most enriched GO BP among discordant DE genes. Click here for file

## Declarations

Publication funding for this article has come from CNR, Pisa.

This article has been published as part of *Theoretical Biology and Medical Modelling *Volume 11 Supplement 1, 2014: Selected articles from the 1st International Work-Conference on Bioinformatics and Biomedical Engineering-IWBBIO 2013. The full contents of the supplement are available online at http://www.tbiomed.com/supplements/11/S1.
