# Supplementary material for: Pathway landscapes and epigenetic regulation in breast cancer and melanoma cell lines
Source: Theor Biol Med Model. 2014 May 7;11(Suppl 1):S8. doi: 10.1186/1742-4682-11-S1-S8 (PMC4108926; doi:10.1186/1742-4682-11-S1-S8)
Supplement: Additional File 1 — AF1 Table 1: The list with the expression values of deregulated genes in both cancer cell lines and the corresponding information extracted from F-census about Gene type: oncogene or TSG (tumor suppressor gene) extracted from both CGC and TSGDB; cancer type according to the classes provided in COSMIC; mutation frequency calculated by six high-throughput mutational screen data of cancer genomes; the number of miRNA predicted to regulate the target cancer genes, extracted from prediction tools including TargetScans, PicTar, DIANA-microT and MirTarget2. AF1 Table 2: Additional information for deregulated genes in MCF7 provided from detection of DNA methylation state in BC. AF1 Table 3: Enriched Gene Ontology Biological processes (GO BP) associated to discordant DE genes. AF1 Table 4: The list of the most enriched functional categories and pathways among the epigenetically modified genes in both cell lines extracted from ConsensusPathDB using ORA tool. AF1 Table 5: The list of TFs found significantly enriched among the epigenetically modified genes in BC and Melanoma. AF1 Figure 1: Graphical representation of the most enriched GO BP among discordant DE genes. [file 1742-4682-11-S1-S8-S1.zip › Additional File 1/AF1 Box of Symbols.docx]

**
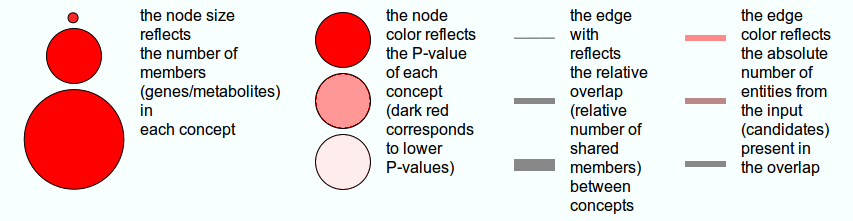
**

**Box 1: symbols for Figure 1 and Figure 2**


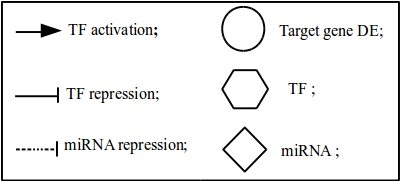


**Box 2: symbols for Figure 3-5**

Node shape, indicating the physical entity (ellipse for epigenetically modified genes, Hexagon for TFs and Diamond for miRNAs); node color, indicating the differentially expressed genes; node border color, indicating the significant TFs enriched (with p-value<0.05) and obtained from the *TFact*S analysis, while the non-colored nodes correspond to TFs that regulate at least one target of the epigenetically modified genes. Then, the label color node reflects the presence of miR-family. The symbols in the Box below are used in the figures.
